# The efficacy of resection of an intradural extramedullary foramen magnum cavernous malformation presenting with repeated subarachnoid hemorrhage: a case report

**DOI:** 10.1186/s13256-017-1220-8

**Published:** 2017-03-09

**Authors:** Tomoya Oishi, Naoto Sakai, Tetsuro Sameshima, Hiroshi Kawaji, Hiroki Namba

**Affiliations:** Department of Neurosurgery, Hamamatsu University School of Medicine, Handayama 1-20-1, Higashiku, Hamamatsu, Shizuoka 431-3192 Japan

**Keywords:** Intradural extramedullary cavernous angioma, Cavernous hemangioma, Foramen magnum, Craniocervical junction, Abducens nerve palsy, Subarachnoid hemorrhage

## Abstract

**Background:**

Intradural extramedullary cavernous angiomas of the central nervous system are a rare type of cavernous angioma, but they can cause fatal subarachnoid hemorrhage. The efficacy of resection for this type of cavernous malformations remains uncertain. This is the first report to recommend surgical resection of these types of lesions regardless of the fatal condition.

**Case presentation:**

Our patient was a 70-year-old Japanese man who experienced a sudden onset of an occipital headache, followed by bilateral abducens nerve palsy. Magnetic resonance imaging revealed a small amount of hemorrhage in both of the lateral ventricles and an intradural extramedullary mass lesion in the left side of his foramen magnum. Two weeks after the appearance of initial symptoms, he became comatose. A computed tomography scan showed an increase in the subarachnoid intraventricular hemorrhaging and of the acute hydrocephalus. Following ventricular drainage, total tumor resection was performed using the lateral suboccipital transcondylar approach in conjunction with a first cervical hemilaminectomy. We observed a grape-like vascular-rich tumor with calcification that was adhering tightly to the wall of his left vertebral artery. A histopathological examination of the surgery specimen identified it as a cavernous angioma. After placement of a ventriculoperitoneal shunt and 2 months of rehabilitation, he recovered completely.

**Conclusions:**

An intradural extramedullary foramen magnum cavernous malformation is quite rare. The fragile surface of our patient’s lesion was causing repeated subarachnoid hemorrhage and consequently progressive fatal neurological deterioration. Surgical resection of the lesion to prevent repeated hemorrhage was performed and he recovered fully. Therefore, we recommend surgical resection of the lesion regardless of the potentially fatal condition.

## Background

Cavernous angiomas are benign vascular lesions and can occur anywhere in the central nervous system (CNS). The majority manifest as intra-axial lesions [1, 2]. The damage caused by the hemorrhaging is usually not fatal; however, cavernous angiomas cause recurrent hemorrhages and hemostasis. Intradural extramedullary cavernous angiomas of the CNS are a rare form of cavernous angiomas. They can cause neurological deficits due to mass effect, cranial nerve palsy, and hemorrhages [1, 3–5]. There are no clinical reviews on extramedullary foramen magnum cavernous malformations. Here we report a case of intradural extramedullary foramen magnum cavernous malformation presenting with repeated subarachnoid hemorrhage (SAH) and progressive neurological deterioration. This is the first report to recommend surgical resection of the lesion regardless of the potentially fatal condition.

## Case presentation

**Fig. 1:**
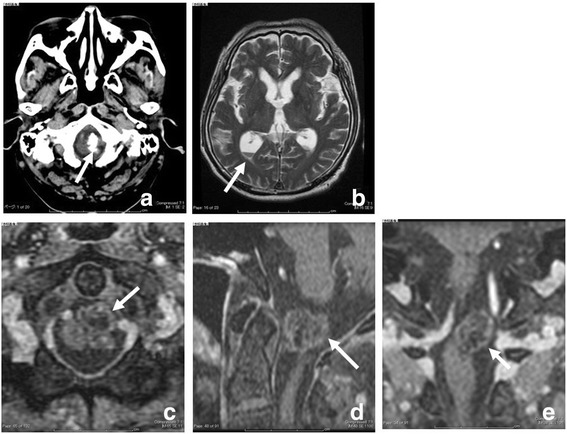
Axial computed tomography demonstrating an intradural extramedullary upper cervical calcified mass lesion (**a**, *white arrow*). Axial T2-weighted magnetic resonance imaging demonstrating an intraventricular hemorrhaging (**b**, *white arrow*). Axial (**c**), sagittal (**d**) and coronal (**e**) T1-weighted contrast-enhanced magnetic resonance images demonstrating an intradural extramedullary mass lesion near the left C1 root which compressed the spinal cord (*white arrows*)

**Fig. 2:**
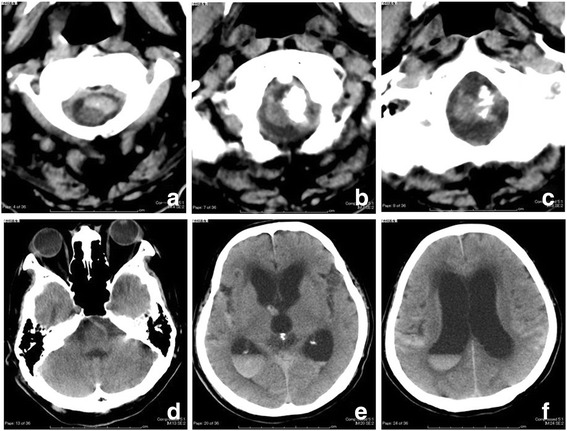
Repeated computed tomography demonstrating hemorrhaging at the ventral side of the cervical calcified lesion (**a**-**c**), as well as subarachnoid, intraventricular hemorrhaging and acute hydrocephalus (**d**-**f**)

A 70-year-old Japanese man experienced a sudden onset of an occipital headache. Two days later, he exhibited bilateral abducens nerve palsy. A neurological examination performed on admission revealed bilateral abducens nerve palsy and mild right-sided paresis. A computed tomography (CT) scan showed there were intradural extramedullary calcified mass lesions on his foramen magnum (Fig. 1a). Magnetic resonance imaging (MRI) showed an intradural extramedullary slightly inhomogeneous enhancing mass (18×16 mm) that spanned his foramen magnum (Fig. 1c-e) and subtle intraventricular hemorrhages in both of the lateral ventricles (Fig. 1b). CT angiography showed no evidence of aneurysms or nidus. He had repeatedly complained of occipital headaches and neck pain. A second MRI revealed an increase in his intraventricular hemorrhages and moderate hydrocephalus. Because the imaging study showed no evidence of threatened herniation or obstructive hydrocephalus, a lumbar puncture was performed. It yielded bloody cerebrospinal fluid indicating SAH. Moreover, the total cell count was elevated owing to an increase in polymorphonucleocytes. A cytological examination revealed no atypical cells. Three days after admission he became comatose, exhibited respiratory disturbance, and tetraparesis. Repeated CT scans showed an increasing SAH and intraventricular hemorrhage as well as acute hydrocephalus (Fig. 2a-f). The mass in the cervicomedullary junction was probably not an aneurysm but a tumor. The initial diagnosis was extra-axial cavernous malformation.

**Fig. 3:**
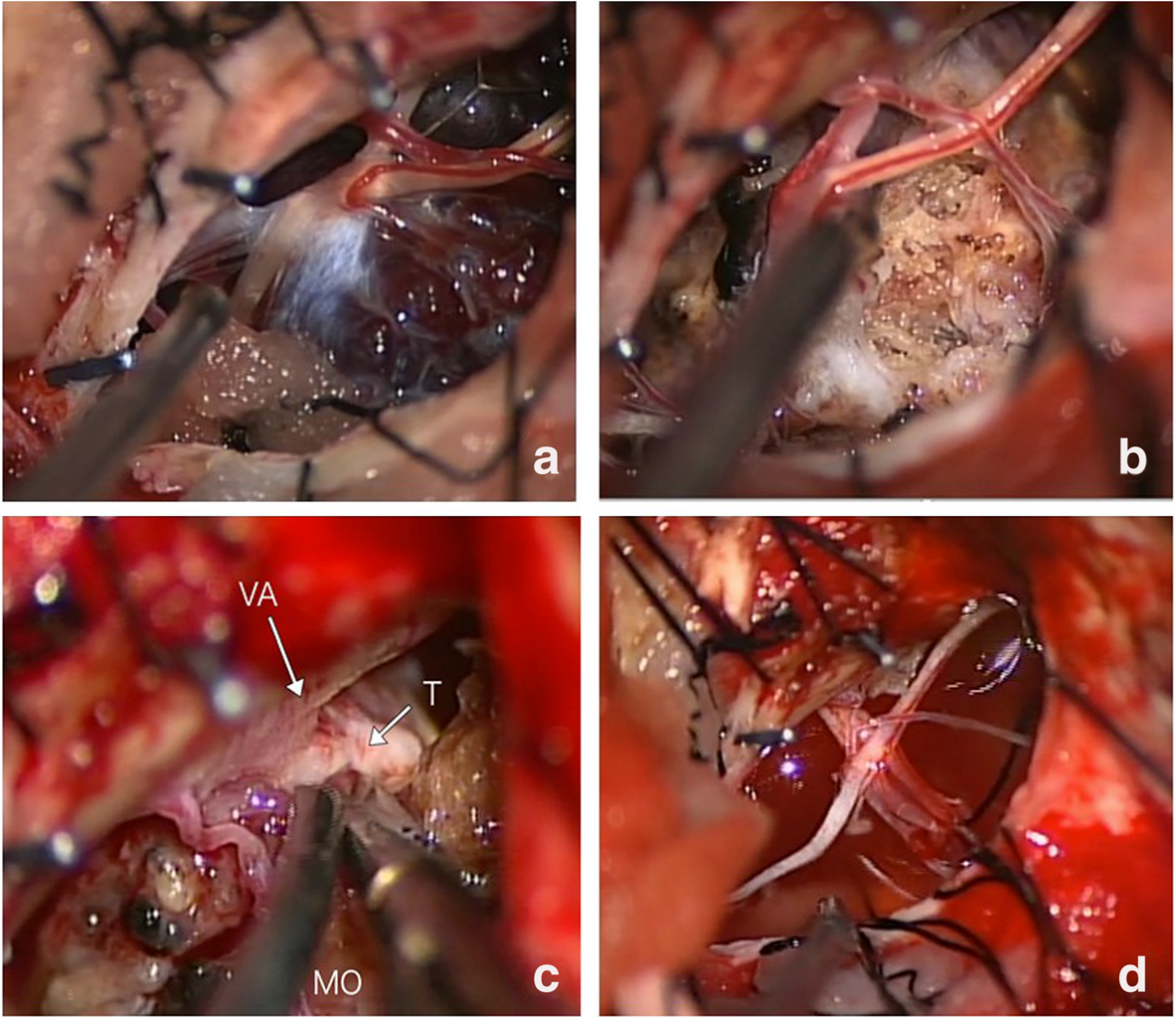
Intraoperative photographs demonstrating an intradural extramedullary mass lesion which has a remarkable vascular grape-like dark red mass (**a**) and the sandy calcification in it (**b**). Although no tight adhesion was found between the tumor and the medulla oblongata, the lesion adhered robustly to the wall of vertebral artery (**c**). Post-tumor resection view, accessory nerve and C1 root were totally preserved (**d**). *MO* medulla oblongata, *T* tumor, *VA* vertebral artery

**Fig. 4:**
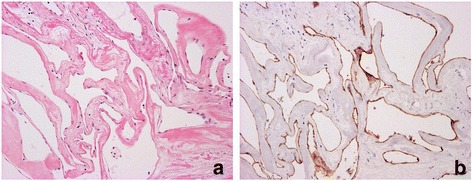
Photomicrographs of the surgical specimen. Dilated vascular spaces of cavernous hemangioma with no elastic lamina and smooth muscle cells. Hematoxylin and eosin, original magnification ×20 (**a**). Immunohistochemical CD31 stain highlighting the mono layer of vascular endothelium, original magnification ×20 (**b**)

After ventricular drainage and respiratory management, we performed a total cervical tumor resection via a lateral suboccipital transcondylar approach in conjunction with a first cervical hemilaminectomy. During surgery, bloody cerebrospinal fluid spilled out of the opening of the arachnoid membrane. Moreover, we observed a grape-like, dark red colored lesion that was highly vascular (Fig. 3a). A slightly organized hematoma was adhering to the ventral side of his spine. The surface of the lesion was very fragile, easily leading to venous hemorrhages. The contents of the mass were remarkably calcificated and sand-like (Fig. 3b). Although the lesion was adhering tightly to his vertebral artery (VA; Fig. 3c), it was detached without damaging the surrounding structures including his cerebellum, medulla oblongata, accessory nerve, and the first cervical root. The lesion was completely removed along with most of the rostral portion of the dentate ligament (Fig. 3d). Postoperative MRI showed that the lesion was completely removed. A histological examination identified the resected tissue as a cavernous malformation (Fig. 4).

His postoperative course was uneventful. He underwent placement of a ventriculoperitoneal shunt for hydrocephalus. Moreover, he showed gradual improvement in his state of consciousness as well as in his bilateral abducens nerve palsy and tetraparesis. After 2 months of rehabilitation, he had fully recovered.

## Discussion

Cavernous angiomas occur in 0.39 to 0.9 % of the general population [3–5]. The natural history of cerebral cavernous angiomas is relatively benign and 10 to 44 % patients are asymptomatic [3, 5, 6]. Some of the frequent clinical manifestations of the disease are seizures, focal neurological deficits, and hemorrhages [3, 5]. The distribution of lesions within the CNS reflects the volume of the various compartments. Most lesions occur in the supratentorial compartment (80 %), followed by the infratentorial compartment (15 %), and then the spinal cord (5 %) [1]. The occurrence of extramedullary cavernous malformations is much lower than that of intramedullary malformations [1, 2, 7, 8]. After a thorough search of the literature on PubMed, we only found three reported cases of an operated intradural extramedullary foramen magnum cavernous hemangioma [9–11]. However, in one case [11] the description of its clinical course was missing. In Table 1, we summarize four cases of extramedullary foramen magnum cavernous malformation including this case. Most of the patients presented with a sudden headache as an initial symptom [9, 10], which was caused by the SAH. Although the preoperative course varied greatly among these cases, the lesion was completely removed, and their outcomes were excellent even though the present case was potentially fatal. Surgical resection is the most effective treatment to prevent recurrence of SAH. Therefore, extramedullary foramen magnum cavernous malformations presenting with SAH should be treated aggressively with surgical resection regardless of their severity.

**Table 1:** Summary of four cases of extramedullary foramen magnum cavernous malformation

No	Author, year	Age, sex	Initial symptoms	SAH	Surgery extent	Origin	Outcome
1	Mocco *et al*., 2005 [9]	21, M	Occipital H/A, photophobia,	Yes	Total	ND	Excellent
2	Palani, 2012 [10]	11, M	Occipital H/A, subtle bilateral corticospinal tract sign	Yes	Total	ND	Excellent
3	Eicker *et al*., 2015 [11]	ND	ND	ND	Total	ND	Excellent
4	Presented case	70, M	Sudden occipital H/A, bilateral abducens nerve palsy, slight right motor deficit	Yes	Total	VA?	Excellent

*F* female, *H/A* headache, *M* male, *ND* not described, *SAH* subarachnoid hemorrhage, *VA* vertebral artery

Previous reports have not described a cavernous malformation originating from the foramen magnum. According to a literature review on spinal cavernous malformations [7], extramedullary cavernous malformations arise from the nerve roots, dura mater, pia mater, and dentate ligament. In this case, we could not determine the origin of the malformation. We found the dark red surface of the lesion through the thin dentate ligament. The lesion was adjacent to the other cerebral structures, and we were able to detach it without cutting the pial surface, the accessory nerve, or the first cervical root. The lesion adhered tightly to the wall of the VA with organized tissue. So, we should take into consideration the wall of the VA as the potential origin of the lesion.

The annual hemorrhage risk rate was determined to range from 0.25 to 3.1 % per patient [3, 4, 6]. The risk of hemorrhage is higher in female patients or in patients with a prior history of hemorrhage [5]. The mechanisms leading to major hemorrhaging remain controversial, given that cavernous hemangiomas are low-flow and low-pressure lesions [12]. In this case, we propose that the lesions’ physical characteristics and its location were contributing factors to inducing hemorrhaging. Operative and histopathological findings confirmed that the surface of the lesion consisted of fragile vessel walls, which can easily cause a hemorrhage. In some cases of spinal extramedullary cavernous malformations [7, 8], SAH may result from the restricted mobility of the lesion. Consequently, the lesion is squeezed between its central calcification and its dynamic surroundings at the foramen magnum.

Although an intradural extramedullary foramen magnum cavernous malformation is very rare, the fragile surface of this lesion was causing repeated SAH. In spite of the potentially fatal condition, our patient recovered fully because recurrence of SAH was prevented; therefore, surgical resection is strongly recommended for intradural extramedullary cavernous malformations. 

## Conclusions

Intradural extramedullary foramen magnum cavernous malformation causes repeated SAH and progressive fatal neurological deterioration. We recommend surgical resection of the lesion to prevent repeated hemorrhage regardless of the potentially fatal condition.

## Abbreviations

CNS: Central nervous system
CT: Computed tomography
MRI: Magnetic resonance imaging
SAH: Subarachnoid hemorrhage
VA: Vertebral artery
